# Developing a novel SARS-CoV-2 risk index to predict the prognostic and therapeutic effects in acute myeloid leukemia

**DOI:** 10.1016/j.heliyon.2023.e22426

**Published:** 2023-11-15

**Authors:** Jiaxin Guo, Yiyi Wei, Qingyan Cen, Jianyu Chen, Yuhua Li

**Affiliations:** a Department of Hematology, Zhujiang Hospital, Southern Medical University, Guangzhou, Guangdong, CN 510280, China; b Bioland Laboratory (Guangzhou Regenerative Medicine and Health Guangdong Laboratory), Guangzhou, Guangdong, CN 510320, China

**Keywords:** SC2RI, AML, Prognosis, Immunotherapy, Drug sensitivity

## Abstract

There is growing evidence of a strong association between SARS-CoV-2 and cancer prognosis and treatment outcome. However, there are no reliable SARS-CoV-2 assessment models to accurately predict prognostic and therapeutic effects in acute myeloid leukemia (AML). Here, differentially expressed genes associated with SARS-CoV-2 were detected, and multiple Cox regression methods were used to construct a SARS-CoV-2 risk index (SC2RI). Then, RT-qPCR was used to validate the gene expression levels in the AML samples. Finally, we explored how the SC2RI affected prognosis, immune infiltration, immunotherapy, and drug sensitivity in AML. We found that CYB5R3 and CLIP4 had been confirmed as hub genes in AML and were used to generate the SC2RI. The datasets indicated that the SC2RI had a superior predictive impact on the prognosis of AML. In addition, high expression of immune checkpoints and numerous immunological infiltrations were substantially correlated with a high SC2RI. However, it responded poorly to immune checkpoint blockade, which may be related to T-cell dysfunction, lack of effective antigens, and deficiency of synaptic capacity. Moreover, a high SC2RI was less susceptible to mTOR-related pathway medications but more sensitive to cell cycle suppressors. Therefore, categorization based on SC2RI could enhance the prognostic prediction of AML and help identify novel therapeutic approaches.

## 1 Introduction

A malignant condition known as acute myeloid leukemia (AML) is linked to aberrant cell formation in hematopoietic stem and progenitor cells. In the bone marrow and peripheral circulation, it is a frequent condition characterized by aberrant dysplasia and hyperplasia of primitive and naïve myeloid cells [1]. According to research by the National Cancer Institute (NCI, 2023) [2], it has been consistently shown that approximately 34 % of all new leukemia types are AML, which develop more frequently with age, especially in those over 70. And most deaths from leukemia are caused by AML, which accounts for 48 % of deaths. In spite of the fact that about 50–70 % of patients with AML achieve remission following routine de novo induction with consolidating chemotherapy, most of them relapse or even die as a result of the treatment [3]. The relative five-year survival rate in AML is simply 30.5 % (https://seer.cancer.gov/statfacts/html/amyl.html). AML is a highly heterogeneous disease associated with numerous gene mutations and cytogenetic abnormalities [4]. The treatment potential of AML is restricted by variations in karyotype, molecular genetics, and clinical presentation in various patients, increasing the need for precision medicine [5]. It is urgent to seek out better therapeutic interventions for AML in light of genomic studies, tumor-targeted medicines, and immunotherapy.

Recently, the worldwide outbreak of Severe Acute Respiratory Syndrome Coronavirus-2 (SARS-CoV-2) has been referred to as the Corona Virus Disease 2019 (COVID-19) pandemic. Along with heart disease and cancer, it now ranks as the third largest cause of death [2], accentuating the problem of inappropriate treatment for people with weakened immune systems, especially cancer patients or the elderly [6]. It has been previously observed that SARS-CoV-2 is more susceptible to resulting in complications in AML [7]. SARS-CoV-2 infection is associated with a mortality rate of 20–52 % in patients with acute leukemia [8], and this rate is higher in those with AML [9]. So far, however, relatively little research has been carried out on the impact of prognosis and therapy in AML with SARS-CoV-2 infection. In order to further determine the extent of the relationship between COVID-19 and AML, bioinformatic analysis were performed to ascertain the biological characteristics and clinical significance of SARS-CoV-2-related genes in AML, as well as their broader applicability to immunotherapy and drug sensitivity.

**Fig. 1:**
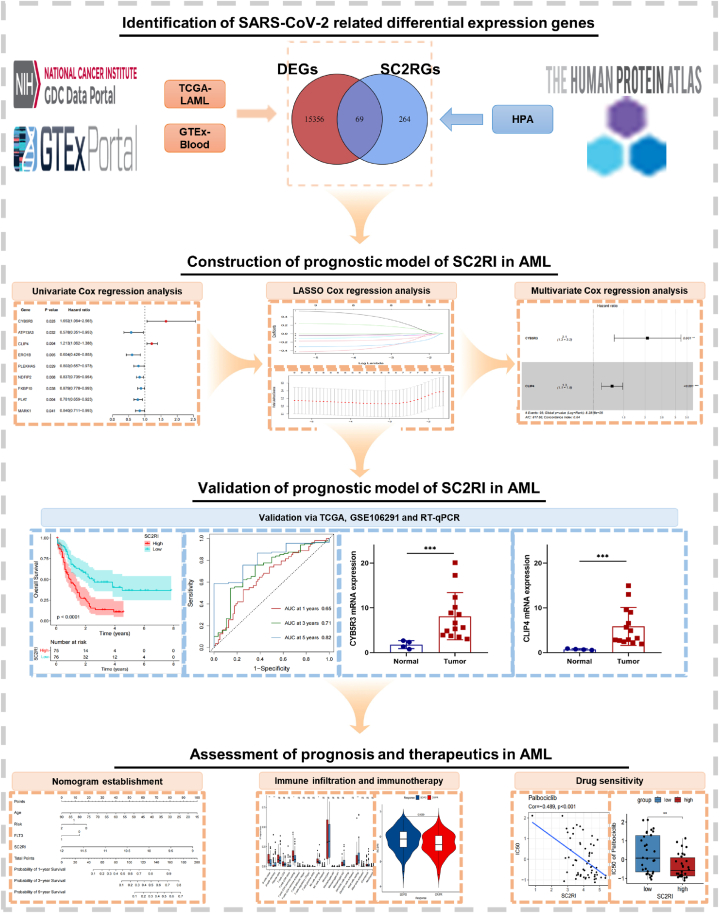
An overview of the research plan. Initially, the differentially expressed genes (DEGs) of the AML tumor and normal groups were analyzed, followed by the identification of SARS-CoV-2 related genes (SC2RGs) from HPA, and SARS-CoV-2 related DEGs were obtained. The core genes were then obtained through various Cox regression analyses to create SC2RI. Subsequently, the model genes were validated using TCGA, GEO, and RT-qPCR. Finally, an investigation was conducted to determine the impact of SC2RI on prognosis, immune infiltration, putative immune escape mechanisms, and drug sensitivity in AML.

This investigation uses existing data from online databases to estimate SARS-CoV-2 associated prognostic genes and construct a novel SARS-CoV-2 related risk model that predicts the prognosis of AML. The study design is depicted in Fig. 1 and Fig. S1. Detailed validation has been carried out in the cohorts of The Cancer Genome Atlas (TCGA) and Gene Expression Omnibus (GEO) [10, 11]. In addition, the expression level of pertinent genes in tumor samples is assessed. Moreover, a nomogram combining pivotal clinical features and risk scores is being developed to estimate the prognosis of AML. In an attempt to offer a fresh perspective on the function of the risk model, the final section seeks to assess the connection between the risk model, biological pathways, immune response, and drug susceptibility in tumors.

## 2 Materials and methods

### 2.1 Data collection

A total of 173 AML samples from TCGA and 337 normal control samples from Genotype-Tissue Expression (GTEx) [12] were downloaded from UCSC Xena [13] and data correction was performed with the “RUVseq” package (k = 2) [14]. Additionally, the “DESeq2” package [15] was utilized to perform an additional differential analysis of the rectified data. According to the standards of adjust p value < 0.05 and |log2FoldChange| ≥ 1, 15425 differentially expressed genes (DEGs) out of 34182 genes were identified, and subsequently visualized using the volcano plot. In addition, 333 SARS-CoV-2 related genes (SC2RGs) were observed in the Human Protein Atlas (HPA) database [16], and 69 SARS-CoV-2 related DEGs were identified by overlapping with the above DEGs. A heat map of gene expression was generated using R software (version 4.2.2) and a protein interaction analysis was carried out on the STRING website [17].

### 2.2 Functional enrichment analysis of SARS-CoV-2 related DEGs

To better understand the functional enrichment of the Kyoto Encyclopedia of Genes and Genomes (KEGG) [18] and Reactome [19] pathways of these 69 SARS-CoV-2 related DEGs, the KEGG Orthology Based Annotation System (KOBAS) database [20] was used for analysis and the corresponding data were thoroughly extracted and mapped using R software. Further, Gene Set Enrichment Analysis (GSEA) [21] was implemented for the Gene Ontology (GO) terms using the “clusterProfiler” package [22]. Meanwhile, the Metascape database [23] was also used for analysis and visualization.

### 2.3 Construction of prognostic model for AML

The clinical characteristics data for AML were taken from UCSC Xena. To construct the model, it was necessary to extract and delete survival data without survival time, and thus 151 data points from AML samples were derived. With univariate Cox regression analysis, 69 genes were analyzed using the “survival” and “survminer” packages, and nine prognostic genes were identified. After that, nine genes were examined using the least absolute selection and shrinkage operator (LASSO) and multivariate Cox regression analysis. Finally, forest maps were generated using the “forestplot” packages. The following step was taken to develop the SARS-CoV-2 risk index (SC2RI) model:

$$\text{SC}2\text{RI}=\sum_{i=1}^{n}\left(\text{Coefficient}\times \text{Expression}\right)$$

### 2.4 Validation of prognostic model of SC2RI

Based on the median of SC2RI, patients were classified into high- and low-risk groups. In order to evaluate the predictive accuracy of the models, Kaplan-Meier (KM) survival curves were analyzed, and receiver operating characteristic (ROC) curves were applied using the “ROCR” [24] and “survivalROC” packages. ROC curves can be used to quantify model quality or predict accuracy by calculating the area under the curve (AUC). In addition to the median, the best Youden's index for five years was calculated using the ROC curves and used for the KM survival analysis. Furthermore, a linkage map was also made based on the model to visualize the correlation between the risk index and the expression level. Finally, the SC2RI model was validated on GSE106291 [25] as well.

### 2.5 Correlation analysis between SC2RI and clinical characteristics

Initially, a comprehensive compilation of clinical trait data in AML was conducted, and NA samples were deleted. In order to evaluate SC2RI levels for relevant clinical features including age, gender, subtype, fms-like tyrosine kinase 3 (FLT3 mutation), nucleophosmin 1 (NPM1) mutation, neoadjuvant treatment, cytogenetic risk, and cytogenetic abnormalities, the “ggplot2” packages were used for visualization, respectively. In addition, the heat map of gene expression was drawn by the “ComplexHeatmap” package [26], and survival time was annotated using scatter plots.

### 2.6 Construction and evaluation of nomogram

To examine the link between survival time and one or more predictors, the SC2RI and other clinical parameters were subjected to both univariate and multivariate Cox regression analysis, and the results were displayed with forest maps and univariate ROC curves. Based on the findings of the regression analysis, age, cytogenetic risk, FLT3 mutation, and SC2RI were integrated to establish a nomogram for forecasting 1-, 3-, and 5-year survival probabilities, and accuracy was evaluated with calibration curves.

### 2.7 Analysis of the relationship between immune infiltration and SC2RI

Based on the median of SC2RI, AML tumor samples were split into two groups with high- and low-SC2RI, and a difference analysis was conducted. Following that, Metascape and GSEA were used for the purpose of identifying putative signaling pathways. Besides, ESTIMATE score was generated by the ESTIMATE algorithms [27] and correlation analysis with SC2RI was carried out. Moreover, marker genes of 28 different immune cells were downloaded from the study by Charoentong P. et al. [28], and the “GSVA” package [29] was employed to identify immune infiltration in single-sample GSEA (ssGSEA). Meanwhile, CIBERSORT algorithms [30] were proposed to quantify the abundance of 22 types of immune cells infiltrating different groups. Finally, it was also attempted to examine the association between SC2RI and the expression levels of 48 common checkpoints and 24 HLA family genes [31, 32].

### 2.8 Assessment of immunotherapy response

Based on the Tumor Immune Dysfunction and Exclusion (TIDE) algorithms [33], the scores for T-cell dysfunction, T-cell exclusion, and TIDE signatures were generated, and a correlation analysis with SC2RI was performed. In addition, in an attempt to verify the relationship between SC2RI and immune checkpoint therapy for PD-1/L1, the “IOBR” package [34] was utilized to capture and analyze data from IMvigor210 [35], GSE135222 [36], and PRJEB25780 [37].

### 2.9 Assessment of drug sensitivity

From the CellMiner database [38], we acquired total information on drug sensitivity and gene expression. After screening medication sensitivity information confirmed by research trials and the Food and Drug Administration, a Pearson correlation analysis was carried out to identify the interaction between SC2RI and drug sensitivity.

### 2.10 RNA extraction and quantitative real-time polymerase chain reaction (RT-qPCR)

Prior to undertaking the investigation, ethical clearance was obtained from Zhujiang Hospital, Southern Medical University. Trizol (Invitrogen) was utilized to extract RNA from the sample in accordance with the manufacturer's instructions, followed by reverse transcription and PCR performed, respectively, with HiScript III RT SuperMix for qPCR (+gDNA wiper) from Vazyme and TB Green® Premix Ex Taq™ II from Takara. The primer sequence of the genes can be found in Table S1.

### 2.11 Statistical analysis

R (version 4.2.2) and GraphPad Prism (version 8.0) were utilized to process the statistical analysis. Analysis of variance and t-tests were performed as needed to determine statistical significance. In addition, a *p* value of 0.05 or less was considered significant (ns, no significance; *, *p* < 0.05; **, *p* < 0.01; ***, *p* < 0.001; ****, *p* < 0.0001).

## 3 Results

### 3.1 DEGs acquirement and functional enrichment analysis

**Fig. 2:**
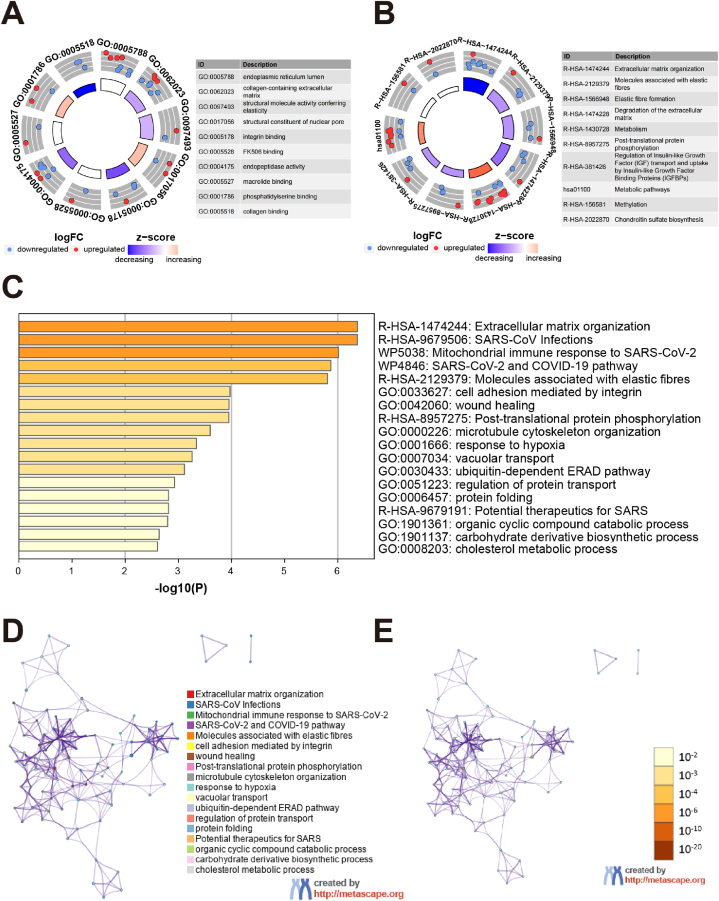
Functional enrichment analysis of 69 SARS-Cov-2 related DEGs. (A) Ten GO terms were performed followed by GO enrichment analysis. (B) Ten KEGG and Reactome terms were performed, followed by a KOBAS enrichment analysis. (C) Significant pathways based on Metascape analysis were performed and clustered, with the network of pathways colored by terms (D) and *p*-value (E), respectively.

Gene expression data among 173 AML patients and 337 normal controls were extracted from the UCSC Xena database, and DEGs were analyzed and visualized (Figs. S2A and B). Based on the overlap of DEGs with 333 SARS-CoV-2 related genes, 69 SARS-CoV-2 related DEGs were revealed (Fig. S2C and Table S2), and a heat map visualization was created (Fig. S2D). These SARS-CoV-2 related DEGs were classified into three categories according to their expression level. Overall, the SARS-CoV-2 related DEGs were screened for protein interactions, and three types were clustered according to K-means clustering, resulting in the construction of a protein-protein interaction (PPI) network (Fig. S2E). It was apparent that these proteins interacted closely with each other. Subsequently, the SARS-CoV-2 related DEGs were subjected to functional enrichment analysis. In terms of GO enrichment analysis, these genes primarily belonged to pathways involving endoplasmic reticulum, integrin binding, phosphatidylserine, and collagen binding (Fig. 2A, Table S3). While the results of KEGG and Reactome revealed that these genes were predominantly enriched in extracellular matrix and metabolism related pathways (Fig. 2B, Table S3). Furthermore, the majority of these genes were linked to the related pathways of SARS-CoV-2, extracellular matrix, protein transport, and folding in the Metascape enrichment analysis (Fig. 2C–E, Table S3).

### 3.2 Construction and verification of SC2RI model

**Fig. 3:**
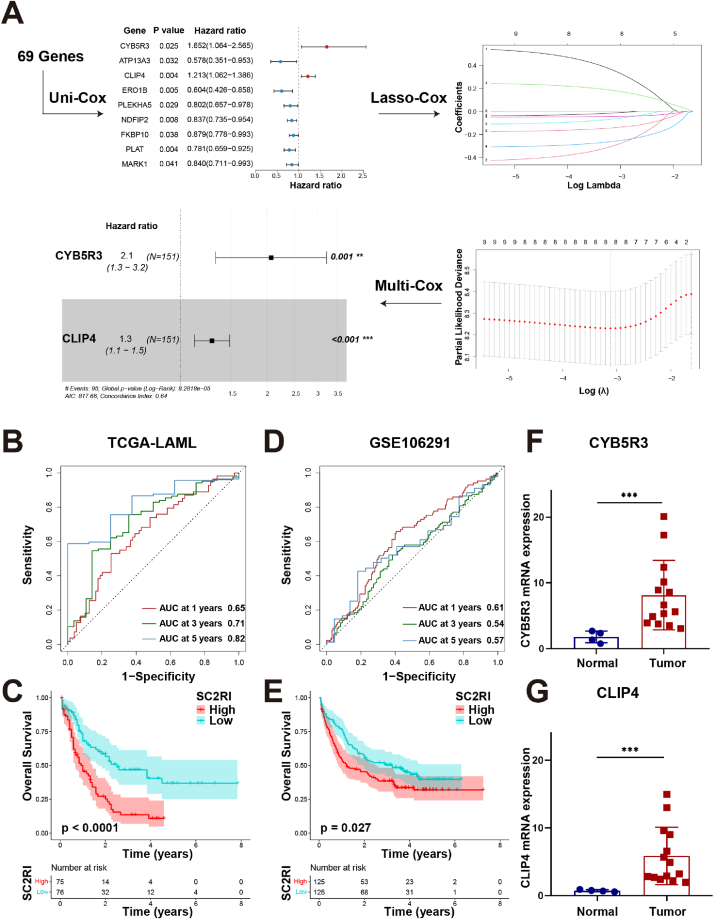
Construction and verification of the SC2RI prognostic model. (A) The acquisition of key genes in a risk model was followed by univariate, LASSO, and multivariate Cox regression analysis. (B) ROC curves for the TCGA-LAML dataset of 1-year, 3-year, and 5-year OS. (C) KM curves for the TCGA-LAML dataset of high- and low-risk groups. (D) ROC curves for the GSE106291 dataset of 1-year, 3-year, and 5-year OS. (E) KM curves for the GSE106291 dataset of high- and low-risk groups. (F, G) RT-qPCR analysis of CYB5R3 and CLIP4 between tumor and normal samples.

Based on expression data and clinical features for AML provided by the TCGA project, we identified nine prognostic genes from 69 SARS-CoV-2 related DEGs by means of univariate Cox regression analysis. Afterwards, two genes, cytochrome *b*5 reductase 3 (CYB5R3) and CAP-Gly domain containing linker protein family member 4 (CLIP4), were further filtered by LASSO and multivariate Cox regression analysis (Fig. 3A). The regression analysis revealed that CYB5R3 and CLIP4 were identified as prognostic risk factors. A risk model described as the SC2RI was established using CYB5R3 and CLIP4 as its foundation.

SC2RI=(0.726×CYB5R3exp)+(0.252×CLIP4exp)

AML samples were split into high- and low-risk groups according to the median SC2RI. A KM survival analysis was conducted, and the accuracy of the predictions was assessed using ROC curves. As displayed in Fig. 3B, the AUCs of 1-year, 3-year, and 5-year overall survival (OS) in the TCGA-LAML were 0.65, 0.71, and 0.82, respectively, indicating that the ROC curves possessed a highly prognostic value. Further KM analysis of the data showed that patients in the high-risk group had a poorer survival probability than those in the low-risk group (Fig. 3C). In addition, we also determined the appropriate cut-off value for the Youden's index in accordance with the 5-year ROC curve. And KM analysis distinguished by the optimal Youden's index demonstrated that patients in the high-risk group had a lesser chance of surviving than those in the low-risk group (Fig. S3A). Based on the model, we created a connection map of risk factors as well. According to the heat map, CYB5R3 and CLIP4 had distinct levels of expression in the high- and low-risk groups, and their levels rose in the former. Meanwhile, patients with high risk had a lower overall survival time than those with low risk (Fig. S3B). The risk model was simultaneously verified in the GSE106291 dataset to assess the robustness of the model, and the analysis outcomes were comparable to those of the TCGA dataset (Fig. 3D, E and Figs. S3C and D). Finally, using RT-qPCR, we validated the expression levels of two key genes in the SC2RI model. In comparison to normal controls, the levels of CYB5R3 and CLIP4 were considerably higher in AML tumors (Fig. 3F and G). Overall, these findings demonstrated that SC2RI had outstanding predictive ability and precisely forecasted the prognosis of AML. Taken together, these results provide important insights into the potential application value of SC2RI.

### 3.3 Correlation analysis between SC2RI and clinical characteristics

**Fig. 4:**
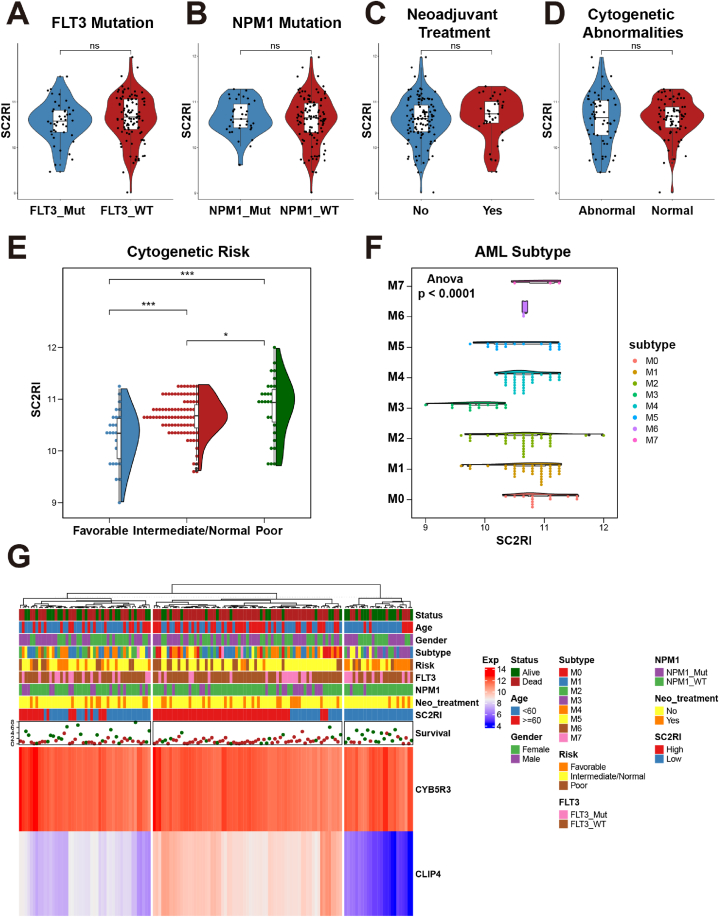
Correlation between SC2RI and clinical features. Violin plots of SC2RI and FLT3 mutation (A), NPM1 mutation (B), neoadjuvant treatment (C), cytogenetic abnormalities (D), cytogenetic risk (E), and AML subtype (F). Heat maps of gene expression, clinical features, SC2RI, and survival data (G).

By compiling clinical features for AML, we examined the distribution of SC2RI in AML patients with various clinical characteristics (Table S4). It was apparent that there was no meaningful disparity between the groups with respect to FLT3 mutation (Fig. 4A), NPM1 mutation (Fig. 4B), neoadjuvant treatment (Fig. 4C), and cytogenetic abnormalities (Fig. 4D). In the cytogenetic risk groups, SC2RI was correspondingly elevated in the poor group compared with the intermediate/normal group and the favorable group (Fig. 4E). In the analysis of AML subtype, SC2RI was significantly lower in type M3 than in the other types (Fig. 4F). According to the heat maps of SC2RI and clinical characteristics, the expression of CYB5R3 and CLIP4 was favorably associated with SC2RI, while cytogenetic risk classification was inversely correlated with SC2RI (Fig. 4G). Taken together, the above results suggest an association between SC2RI and clinical traits. And AML patients can be distinguished by SC2RI based on the major markers mentioned above.

### 3.4 Establishment and evaluation of nomogram

**Fig. 5:**
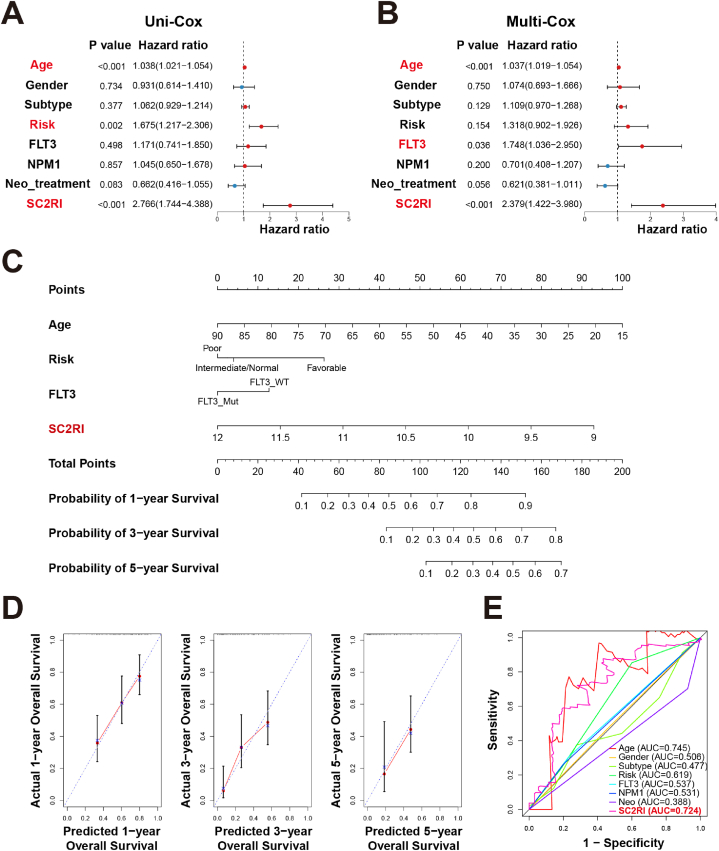
Establishment and evaluation of the nomogram. Forest maps of SC2RI and other clinical parameters in AML were analyzed by univariate (A) and multivariate (B) Cox regression analysis. (C) Nomogram for prediction of 1-, 3-, and 5-year survival probabilities in AML. (D) Calibration curves of the nomogram. (E) ROC curves of SC2RI and clinical parameters.

In an effort to better understand the connection between clinical characteristics, SC2RI, and prognosis, we employed clinical features and SC2RI for univariate and multivariate Cox regression analysis. Age, cytogenetic risk, and SC2RI were considered prognostic risk factors for AML in the univariate Cox regression analysis (Fig. 5A). While age, FLT3 mutation, and SC2RI were prognostically detrimental elements for AML in multivariate Cox regression analysis (Fig. 5B). The most striking observation that emerged from the regression analysis was that SC2RI was an independent prognostic factor for AML. Therefore, a nomogram was created to assess the probability of survival of an AML patient over 1, 3, and 5 years based on their age, cytogenetic risk, FLT3 mutation, and SC2RI (Fig. 5C). Strong evidence from calibration curves demonstrated that Nomogram had a better capacity for prediction (Fig. 5D). In addition, the predictive performance of the clinical features and SC2RI was assessed using ROC curves. The top three AUCs were as follows: the AUC of age was 0.745, the AUC of SC2RI was 0.724, and the AUC of cytogenetic risk was 0.619 (Fig. 5E), indicating that SC2RI had greater predictive value.

### 3.5 Association of SC2RI with immune aspects in AML

**Fig. 6:**
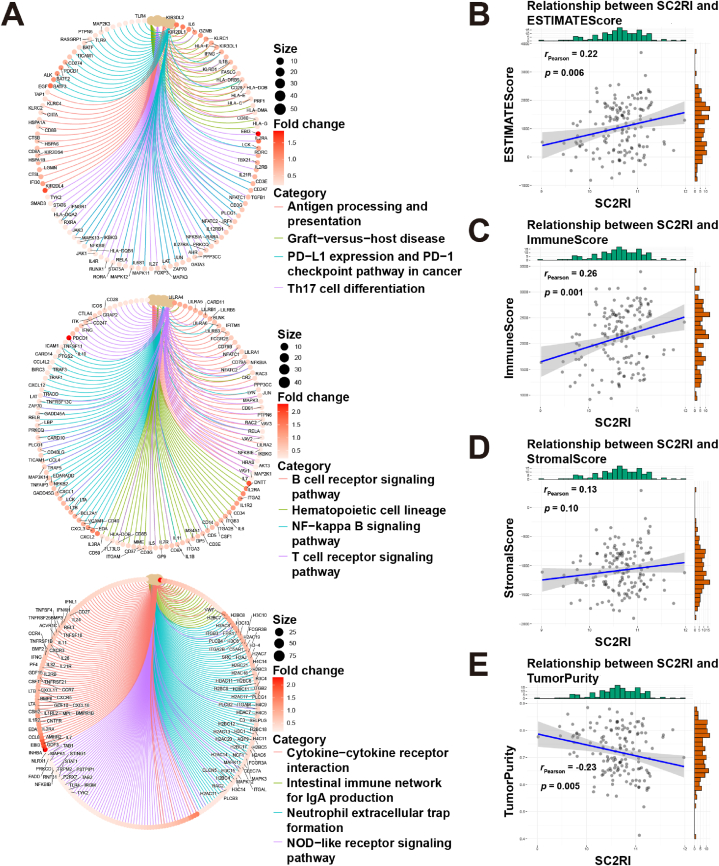
GSEA analysis and ESTIMATE algorithms among different groups of SC2RI in AML. (A) Signaling functions and pathways of biological processes performed with GSEA. Correlation analysis between SC2RI and ESTIMATE score (B), immune score (C), stromal score (D), and tumor purity (E).

**Fig. 7:**
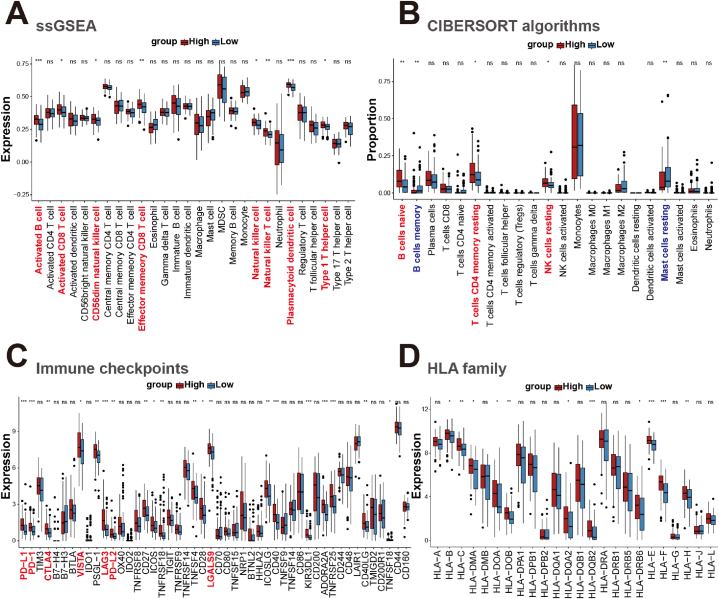
Comparative analysis between immune infiltration, immune checkpoints, HLA family and SC2RI in AML. Analysis of immune cell infiltration between high- and low-groups of SC2RI using ssGSEA (A) and CIBERSORT algorithms (B). Expression levels of immune checkpoints (C) and HLA family genes (D) differed between high- and low-groups of SC2RI.

The relationship between SC2RI and AML immune signaling was then further investigated. First, they were separated into two groups with low and high median values of SC2RI, and Metascape (Table S5) and GSEA (Table S6) were run to analyze the major signaling pathways. In Metascape analysis, the top 20 pathways were shown in Fig. S4A, and cluster (Figs. S4B and C) and module (Fig. S4D) analysis were constructed. The results demonstrated that DEGs were particularly abundant in EGFR, GPCR, ECM, and integrins, as well as in synaptic pathways. In terms of GSEA, innate immune pathways, antigen presenting and processing pathways, immune checkpoint related pathways, and immune cell receptor signaling pathways were already activated in patients with high SC2RI (Fig. 6A), which revealed a potential link between SC2RI and immune signaling. Furthermore, SC2RI was positively correlated with ESTIMATE score (Fig. 6B) and immune score (Fig. 6C), which were negatively correlated with tumor purity (Fig. 6E). However, the stromal score did not seem to be affected by SC2RI (Fig. 6D). The results of the ESTIMATE algorithms illustrated that the amount of infiltrating immune cells increased and tumor purity decreased in the high SC2RI group. In addition, we examined immune infiltration (Fig. 7A and B), immune checkpoints (Fig. 7C), and HLA family gene expression (Fig. 7D) in high and low SC2RI groups. The results indicated that infiltration of activated B-cells and CD8^+^ T cells, effector memory CD8^+^ T cells, and natural killer (NK) cells were abundant in the high SC2RI group. Meanwhile, numerous common immune checkpoints such as PD-1/PD-L1, CTLA4, VISTA, LAG3, and LGALS9 were elevated, and levels of HLA family genes were also increased in the high SC2RI group.

### 3.6 Assessment of immunotherapy response based on SC2RI

**Fig. 8:**
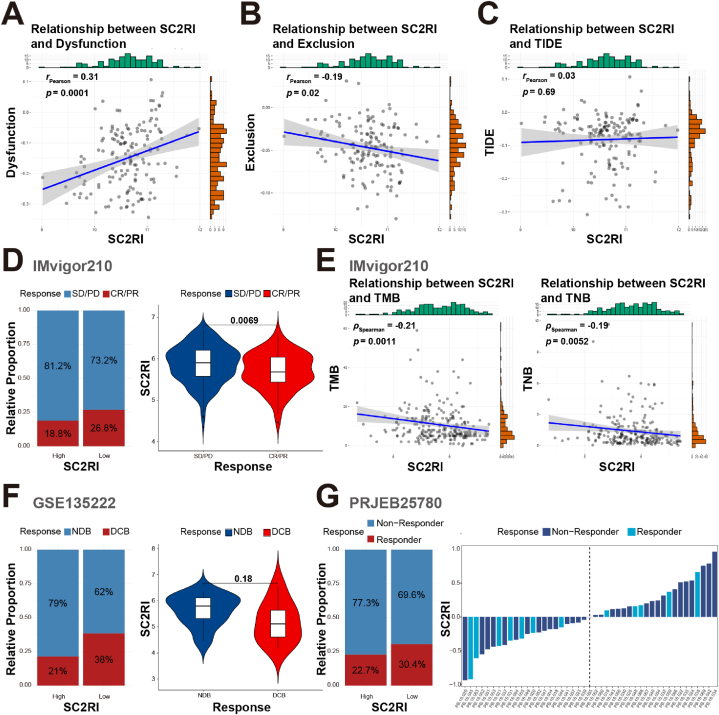
Assessment between immunotherapy response and SC2RI. Correlation analysis between TIDE and SC2RI for dysfunction scores (A), exclusion scores (B), and TIDE scores (C). (D) Comparison of immunological efficacy of immunotherapy between high- and low-groups of SC2RI in IMvigor210. (E) Correlation analysis between SC2RI, TMB, and TNB. Other relative immunoefficacy differences in immunotherapy were observed between high- and low-groups of SC2RI in GSE135222 (F) and PRJEB25780 (G). SD, stable disease; PD, progressive disease; CR, complete response; PR, partial response; NDB, no durable benefit; DCB, durable clinical benefit.

As mentioned in the results above, T cells and NK cells were enriched in the group with high SC2RI, and the levels of immune checkpoints were up-regulated as well. Therefore, TIDE algorithms were implemented to anticipate the tumor response to immunotherapy. It might be accurate to predict that patients with a high correlation were those who did not respond to immunotherapy by correlating the expression data of tumor patients with T-cell dysfunction scores and T-cell exclusion scores, separately. As presented in Fig. 8, SC2RI was positively correlated with T-cell dysfunction (Fig. 8A) and negatively correlated with T-cell exclusion (Fig. 8B), but no statistically significant correlation was observed in TIDE (Fig. 8C). Since higher levels of T cell infiltration were linked to high SC2RI, T cell dysfunction could be the cause of resistance to tumor immunotherapy. In addition, considering that there was no concrete and detailed dataset on AML with immunotherapy, IMvigor210 (urothelial carcinoma), GSE135222 (non-small cell lung cancer), and PRJEB25780 (metastatic gastric cancer) trials were used to scrutinize the capacity of SC2RI to forecast the responsiveness of immune checkpoint blockade (ICB). In the IMvigor210 dataset, surprisingly, ICB immunotherapy produced a lower percentage of CR/PR (complete response/partial response) in the high SC2RI group in comparison with the low SC2RI group. The SC2RI of the CR/PR group was lower than that of the SD/PD (stable disease/progressive disease) group, indicating that the immunotherapy effect in the high SC2RI group might be unsatisfactory (Fig. 8D). In addition, both tumor mutation burden (TMB) and total neoantigen burden (TNB) were predictive markers of ICB efficacy. Correlation analysis of TMB and TNB demonstrated a negative association between SC2RI and both TMB and TNB (Fig. 8E). Additionally, in the GSE135222 and PRJEB25780 cohorts, the high SC2RI group displayed a higher no-durable benefit and non-respond ratio (Fig. 8F and G). These results indicated that the high SC2RI group was more likely to be ineffective in ICB treatment.

### 3.7 Assessment of drug sensitivity based on SC2RI

**Fig. 9:**
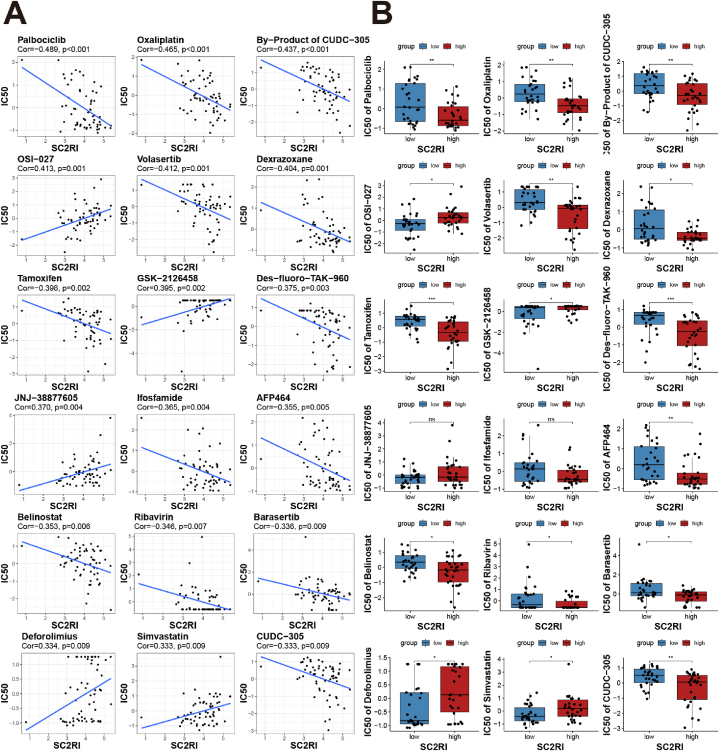
Correlation and comparison analysis between drug sensitivity and SC2RI. (A) Identify the interaction between SC2RI and drug sensitivity by Pearson correlation in 18 anti-tumor medications. (B) Comparison of the IC50 differences between high- and low-groups of SC2RI in 18 anti-tumor medications.

In order to look for promising therapeutic medications, we further probed into the link between SC2RI and drug sensitivity in CellMiner. As depicted in Fig. 9, we gathered relevantly meaningful information between SC2RI and 18 medications, which included CDK4/6 inhibitors (Palbociclib), antitumor drugs (Oxaliplatin, Ifosfamide, and Belinostat), adjuvant antitumor drugs (Dexrazoxane), HSP90 inhibitors (CUDC-305, By-Product of CUDC-305), mTOR inhibitors (OSI-027, GSK-2126458, Deforolimus), PLK1 inhibitors (Volasertib, Des-fluoro-TAK-960), estrogen receptor antagonists (Tamoxifen), c-Met inhibitors (JNJ-38877605), HIF-1α inhibitors (AFP464), Aurora B inhibitors (Barasertib), antiviral agent (Ribavirin), and hypolipidemic drugs (Simvastatin). The detailed drug information can be seen in Table S7. Most medications, including drugs that could inhibit cell cycle (Palbociclib, Volasertib, TAK-960), HSP90 inhibitors (CUDC-305), and HIF-1α inhibitors (AFP464) seemed to have a negative association between SC2RI and their half maximal inhibitory concentration (IC50) values. In other words, these drugs are more sensitive in the high SC2RI group. Notably, the IC50 values of mTOR pathway inhibitors (OSI-027, GSK-2126458, and Deforolimus) displayed a favorable correlation with SC2RI, indicating that the drug sensitivity of mTOR inhibitors was worse in high SC2RI group.

## 4 Discussion

Since the COVID-19 pandemic, research has revealed that cancer patients have higher fatality rates and are more susceptible to the virus [39]. An independent predictive risk factor for COVID-19, particularly in the elderly, is malignant disease [40]. Cancer patients with weakened immune systems bear a high burden of severe COVID-19 [6] because the virus impairs immune response and inflammation [41]. Regarded as one of the most prevalent hematological cancers, AML has a terrible prognosis and no reliable prognostic markers. AML impedes the maturation of white blood cells, leading to the accumulation of immature cells in the bone marrow, their expansion, and the suppression of hematopoietic production. A key factor contributing to the poor prognosis of AML is immunodeficiency. T- and NK cells are the main immune cell types that manifest immunodeficiency in AML [42]. The major factors contributing to immunological dysfunction in AML are excessive maturation of NK cells, and senescence and depletion of T cells. These factors lead to immunosuppression, which makes it easier for leukemia to evade the immune system [43]. There are case reports of malignant primitive cell elimination and clinical remission in COVID-19 infected individuals with AML after supportive treatment [44], despite several studies showing an increased mortality rate in AML patients infected with COVID-19 [9]. This sparked our interest in undertaking research related to SARS-CoV-2 and AML.

In order to formulate a predictive model for the prognosis and therapeutic effectiveness of AML patients, a total of two genes (CYB5R3, CLIP4) were assessed following Cox regression in this research. Previous studies have linked CYB5R3 and CLIP4 to cancer diagnosis and prognosis. CYB5R3, a gene involved in amino acid metabolism and controlling oxidation-reduction (redox) processes at the subcellular level, is a member of the CYB5R family [45]. It is now known that CYB5R3 mutations are the cause of methemoglobinemia [46]. In addition, CYB5R3 is believed to be an oncogene, and its overexpression is intertwined with poor prognosis in breast cancer and hepatocellular carcinoma [47, 48]. Some cancers may be sheltered from oxidative stress and apoptosis if CYB5R3 is overexpressed in these tumors [47, 49]. Furthermore, it is also thought that the tumor protein CLIP4 is a carcinogenic molecule. As a promising molecule for predicting metastasis, increased expression of CLIP4 in renal cell carcinoma may dramatically increase cell viability and migration [50]. CLIP4 methylation can be used as a biomarker to identify and screen for early colorectal and gastric malignancies. The level of CLIP4 methylation in cancer tissues was much higher than in adjacent tissues [51, [52]. Highly methylated CLIP4 can be found in circulating tumor DNA and employed as a surrogate molecule to enhance the accuracy of clinical cancer diagnosis [52, 53]. Furthermore, in this study, we discovered that CYB5R3 and CLIP4 were substantially elevated in AML, indicating a poor prognosis. However, there is minimal information in the literature on the relationship between CYB5R3, CLIP4, and AML. As a consequence, to better understand the precise contributions and probable processes of CYB5R3 and CLIP4 in AML, additional experimental studies are urgently required.

Using two DEGs (CYB5R3 and CLIP4), we constructed the SC2RI model for AML. Based on the median and ideal Youden's index of SC2RI, patients in the TCGA-LAML dataset were separated into high- and low-risk groups. Significant differences existed between the two groups in the pattern of gene expression, probability and duration of survival. The ROC curves also demonstrated that SC2RI can accurately estimate the survival probability of AML patients. The GSE106291 dataset was utilized to further validate the model. SC2RI was an independent risk factor for the prognosis of AML, according to both univariate and multivariate Cox regression analysis. Using conventional molecular and cytogenetic methods for risk stratification [54], SC2RI was shown to be positively connected with genetic risk, with favorable groups tending toward lower SC2RI and poor groups tending toward higher SC2RI. Besides, the ROC curves also highlighted that SC2RI had a superior AUC for predictive accuracy (0.724) than genetic risk stratification (0.619). Subsequently, using clinical variables such as age, cytogenetic risk, FLT3 mutation, and SC2RI, we established a nomogram that has the potential to predict the OS of AML. The innate immunity pathways, immune cell receptor pathways, and immune checkpoint pathways were predominantly stimulated in the high-risk group, according to the GSEA, confirming that there were variances in cell biological processes between the high- and low-risk groups. Further assessment of differences in tumor immune status between the two groups revealed a positive association between SC2RI and various immunosuppressive checkpoints as well as the penetration of activated killer immune cells. Moreover, studies using TIDE scores and immunotherapy datasets revealed that patients with low SC2RI saw a higher proportion of remission or response than those with high SC2RI. In other words, patients with low SC2RI were more likely to benefit from immunotherapy than patients with high SC2RI. This is intriguing as it seems to be at odds with the potential immunotherapeutic effects of extensive immune cell infiltration and high immune checkpoint expression. According to the results of this study and an investigation of the relevant literature, there are primarily three reasons, as follows: (1) Ineffective therapies are linked to immunosuppressive pathways. T-cell dysfunction is a state of inadequate response characterized by increased expression of inhibitory receptors, decreased production of effective cytokines, and diminished cytotoxic activity [55]. Therapies that interrupt the anti-PD-1/L1 pathway allow T cells to resume their typical attacking capabilities. Unfortunately, there is more than one immunosuppressive pathway involved in tumors, and inhibitory checkpoints such as CTLA-4, LAG-3, and LGALS9 also elevate in response to SC2RI, which impairs T-cell activity. Consequently, ICB is completely useless when used alone [56]. (2) Inadequate treatments are associated with antigen loss. Since only effective antigens can trigger immune cells to elicit an anti-tumor immune response, it is currently thought that neoantigens have a considerably greater capacity to activate T-cell than comparable antigens [57]. Patients with tumors that have substantial TMB and TNB possess robust neoantigens, which is a critical indicator of successful immunotherapy [58]. However, in this study, the high SC2RI group appeared to lack useful antigens, and immunotherapy with ICB resulted in unsatisfactory outcomes as TMB and TNB were adversely linked with SC2RI. (3) Unsuccessful treatments related to impaired synaptic capacity of the immune system. In this work, it was discovered that the high SC2RI group had elevated amounts of T-cell infiltration and activated T-cell receptor signaling pathways. The actin-mediated cytoskeletal signaling pathways of integrin, GPCR, and protein-protein interactions at synapses were considerably affected by DEGs between high and low SC2RI. These features may indicate that T cells are less able to form immunological synapses with tumor cells [59, 60], potentially impacting the therapeutic efficacy of ICB. Last but not least, we examined the relationship between SC2RI and drug sensitivity. It was discovered that increased SC2RI was more susceptible to cell cycle inhibitors, but not to drugs related to the mTOR pathway. These results could have certain clinical and therapeutic implications.

Nevertheless, this study still contains a few limitations. Firstly, we build a predictive model using a range of regression techniques and open databases, thus we must accept that the mathematical model may have significant limits relating to its predictive power. Hence, to determine whether the SC2RI model can become an accurate predictor of prognosis and immunotherapy in AML, further review of clinical data is needed, as the database lacks representation in terms of timeliness, sample size, and variances in clinical features. In addition, molecular studies need to be carried out following the results of the enrichment analysis from the database. Besides, since there are almost no samples with ICB therapy in AML, this study utilizes samples from other tumors instead. Therefore, additional collection of AML samples is required to verify the prediction of the model on the effects of immunotherapy. Furthermore, the function and specific mechanism of SC2RI related genes in AML remain unclear and need to be further explored.

In summary, we established a model incorporating two genes to forecast the prognosis of AML, the efficacy of immunotherapy, and the drug sensitivity based on SARS-CoV-2-related genetic material from HPA and publicly available genetic information from the TCGA database. The predictive capability of the model was also verified in the GEO project. It was clearly demonstrated that SC2RI may have prognostic value in AML and is additionally tightly linked to the immunological landscape. Due to dysfunction of T-cell, a shortage of effective antigens, and impaired immune synaptic development, immunotherapy might be less effective in patients with high SC2RI. Meanwhile, increasing SC2RI was less responsive to drugs related to the mTOR pathway and was more sensitive to cell cycle inhibitors. The findings of this research may accurately forecast the prognosis for AML and propose possible therapeutic directions.

## Funding statement

This work was supported by the 10.13039/501100001809National Natural Science Foundation of China [grant number U2001224] and Bioland Laboratory, China (Guangzhou Regenerative Medicine and Health Guangdong Laboratory) [grant number 2018GZR110105014].

## Data availability statement

All data presented in this study are included in the article/supplementary material.

## CRediT authorship contribution statement

**Jiaxin Guo:** Writing – review & editing, Writing – original draft, Visualization, Software, Resources, Methodology, Investigation, Formal analysis, Data curation. **Yiyi Wei:** Writing – original draft, Visualization, Validation, Software, Methodology. **Qingyan Cen:** Writing – review & editing, Software, Resources. **Jianyu Chen:** Writing – review & editing, Validation. **Yuhua Li:** Writing – review & editing, Supervision, Project administration, Funding acquisition, Conceptualization.

## Declaration of competing interest

The authors declare that they have no known competing financial interests or personal relationships that could have appeared to influence the work reported in this paper.
