# Supplementary material for: Developing a novel SARS-CoV-2 risk index to predict the prognostic and therapeutic effects in acute myeloid leukemia
Source: Heliyon. 2023 Nov 15;9(11):e22426. doi: 10.1016/j.heliyon.2023.e22426 (PMC10700646; doi:10.1016/j.heliyon.2023.e22426)
Supplement: Multimedia component 1 [file mmc1.docx]

**Supplementary material**

**Supplementary Figures: Figure S1-Figure S4**


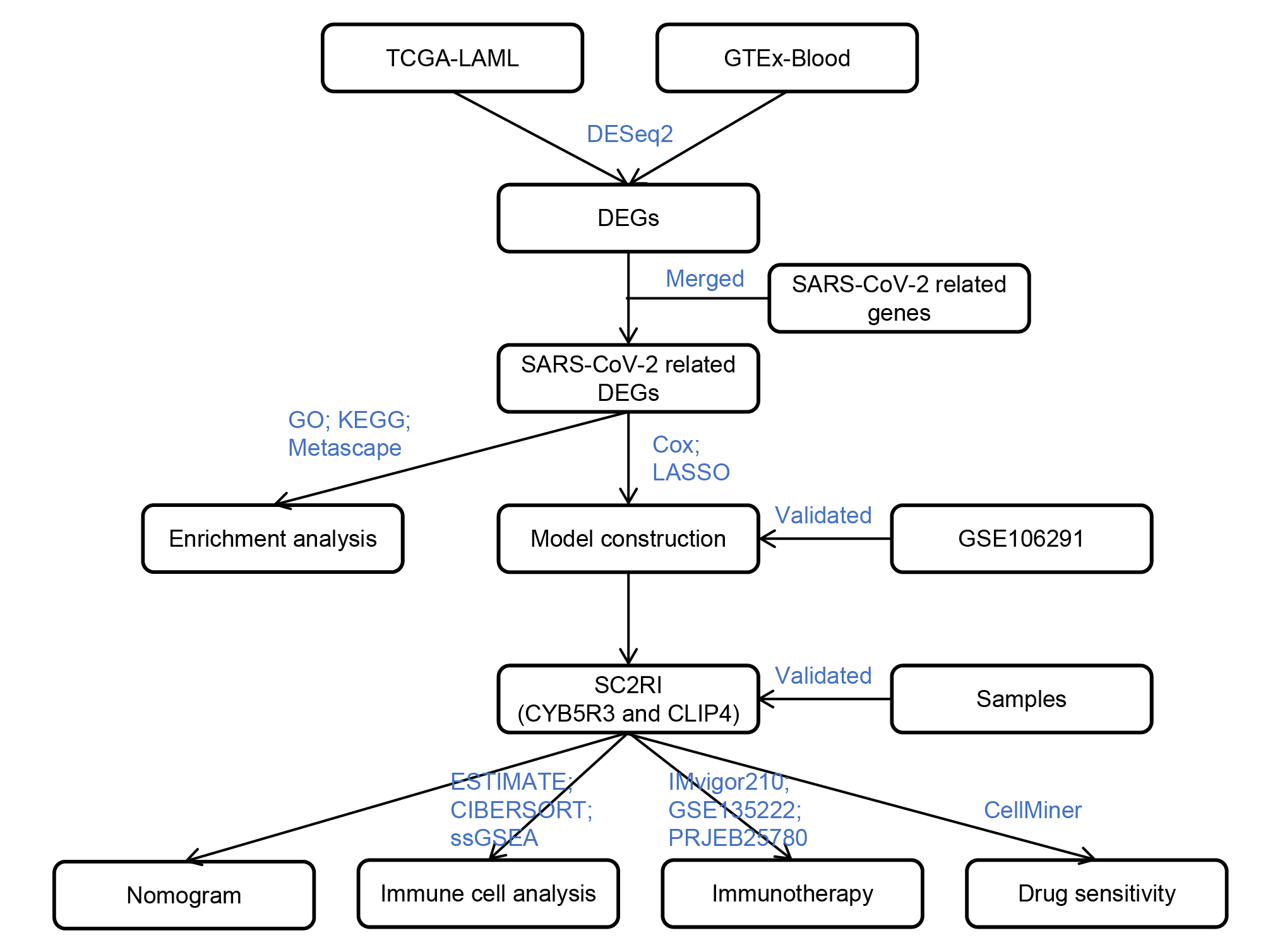
Figure S1. The relevant datasets and a flow chart of the study.


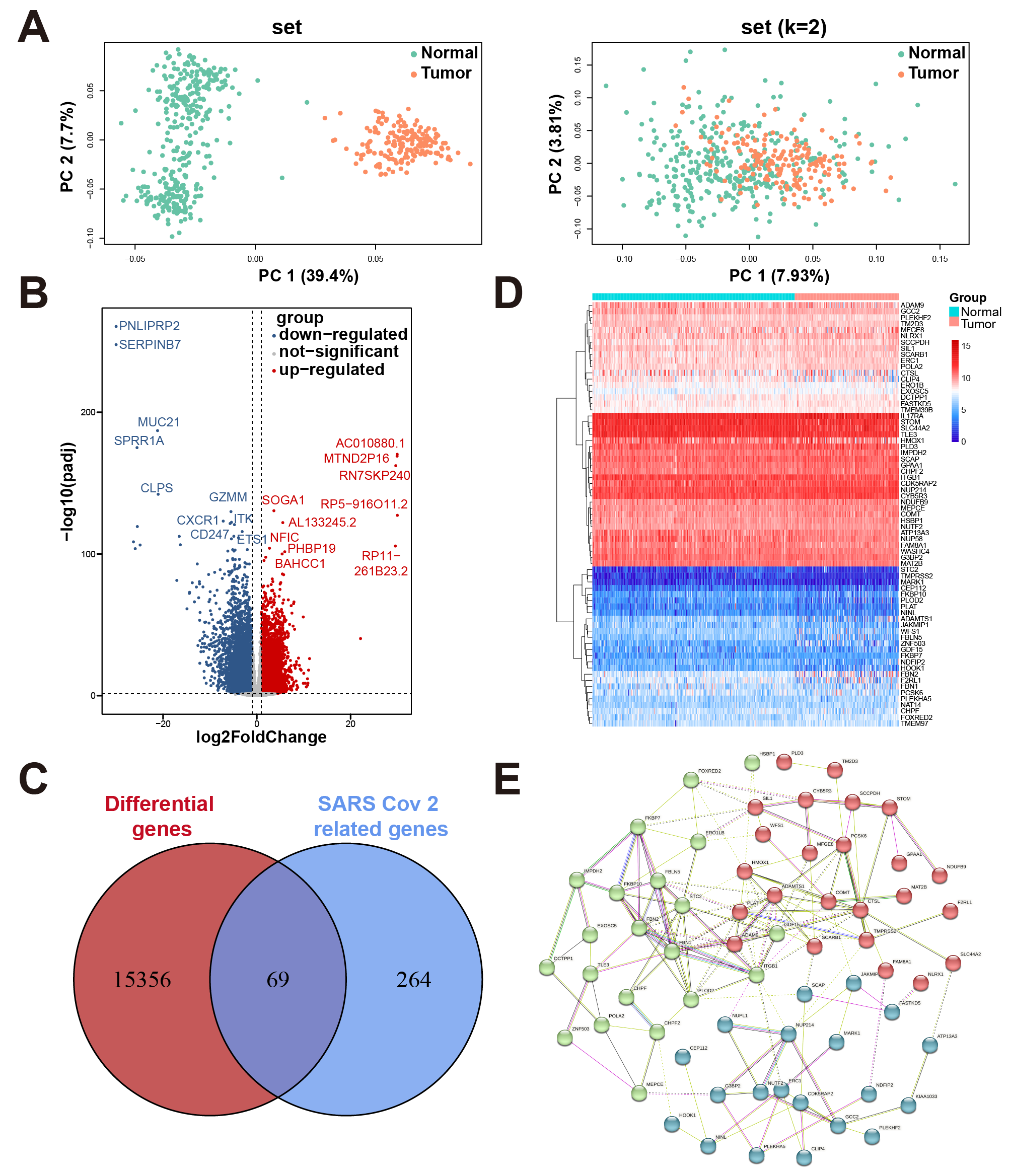


Figure S2. Extraction of SARS-CoV-2 related DEGs and analysis of the PPI network. (A, B) Principal component analysis of 173 AML patients and 337 normal controls based on different adjusted k values. (B) The volcano plot of DEGs between tumor and normal samples. (C) Overlap of DEGs and SARS-CoV-2 related genes. (D) The heat map of SARS-CoV-2 related DEGs. (E) PPI network was performed with the STRING tool and divided into three modules.


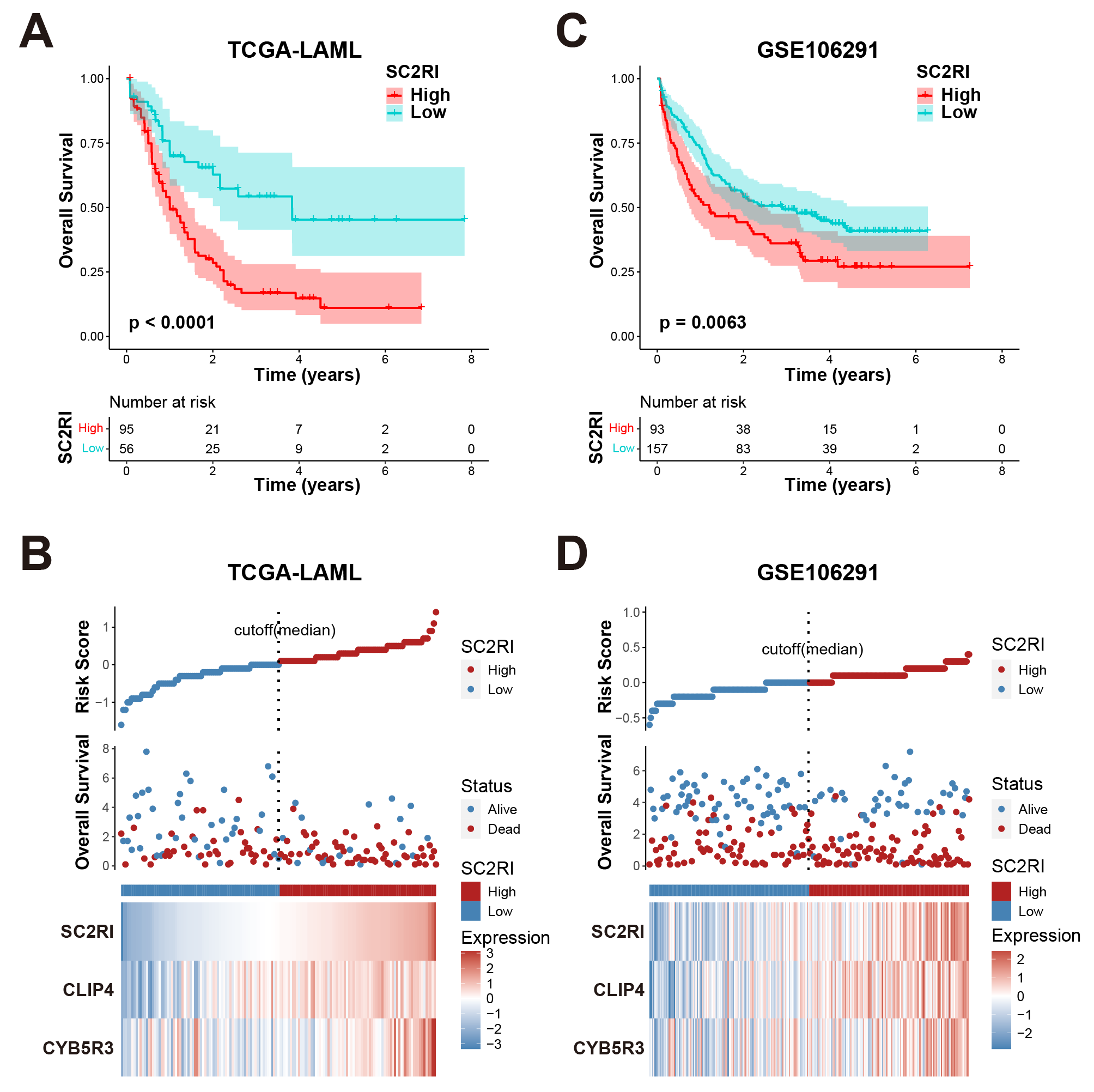


Figure S3. Validation of SC2RI prognostic model. KM curves for the high- and low-risk groups cutoff by the ideal Youden's index for the TCGA-LAML (A) and GSE106291 (C) datasets. Diagram illustrating the relationship between risk factor scores, gene expression levels, and patient survival time in the TCGA-LAML (B) and GSE106291 (D) datasets of high- and low-risk groups cutoff by median.


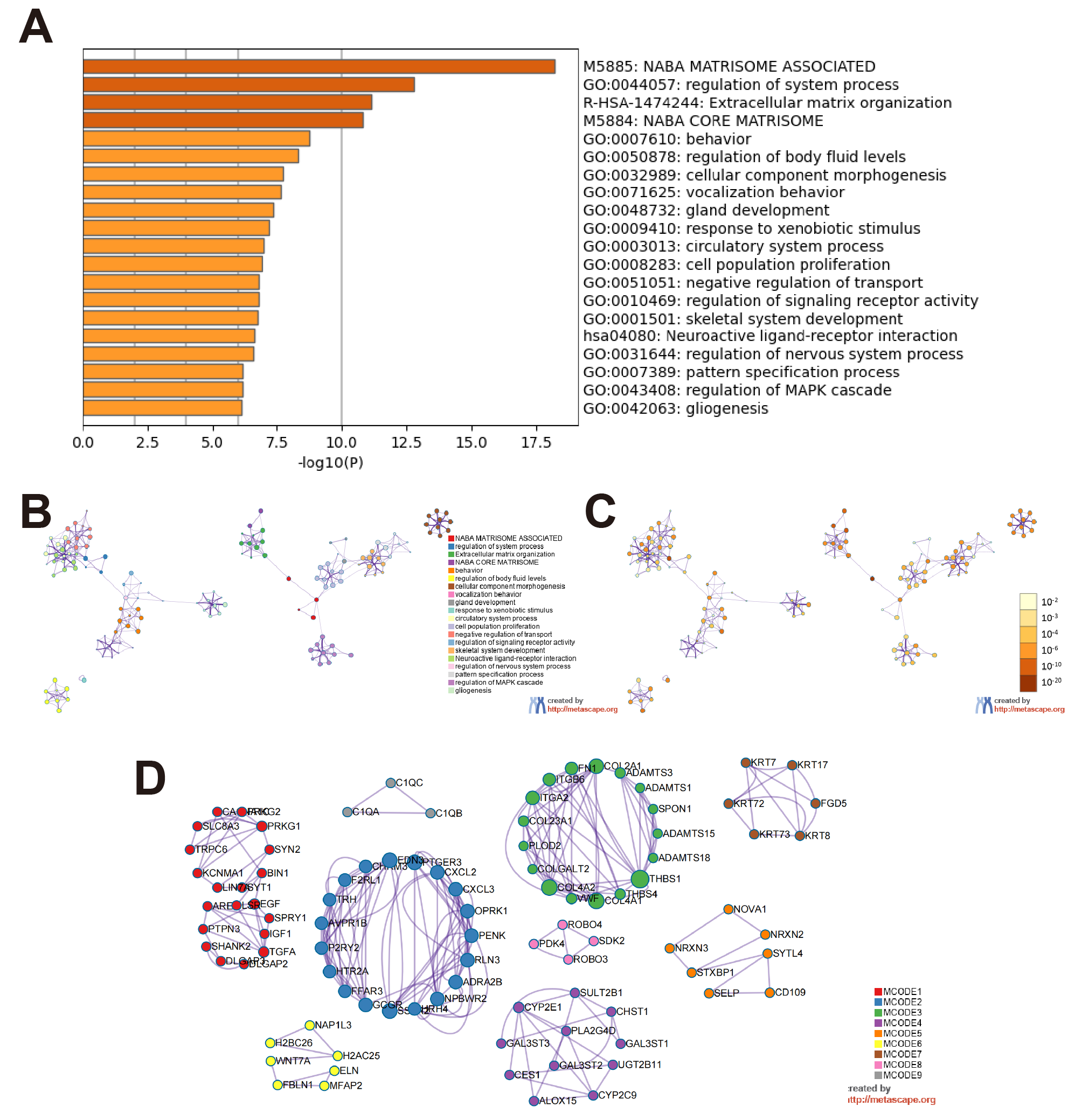


Figure S4. Functional enrichment analysis (Metascape) among different groups of SC2RI in AML. (A) The top 20 enriched terms illustrated by bar plots. (B) Enrichment cluster and network by representative terms. (C) Enrichment cluster and network by p-value, and statistical significance of the node increased with color darkness. (D) Identify proteins with densely connected neighborhoods and protein-protein interactions networks by the MCODE algorithm.

**Supplementary Tables: Table S1-Table S7**

Table S1. The primer sequence of genes for RT-qPCR.

Table S2. 69 SARS-CoV-2 related differentially expressed genes.

Table S3. The results of functional enrichment analysis for SARS-CoV-2 related DEGs.

Table S4. Clinical characteristics of AML patients from UCSC Xena.

Table S5. The results of Metascape for SC2RI in AML.

Table S6. The results of GSEA for SC2RI in AML.

Table S7. The results of drug sensitivity and SC2RI in CellMiner.

**Supplementary Tables: Table S1-Table S7**

**Table S1.** The primer sequence of genes for RT-qPCR.

| Gene | Species | Primer Sequence |
| --- | --- | --- |
| CYB5R3 | Human | Forward: 5’-TTGCCAACCAGACCGAGAAGGA-3’ |
|  |  | Reverse: 5’-CCAGCGTGTACCAGAGCTTGAAG-3’ |
| CLIP4 | Human | Forward: 5’-GCTTCAGGTATCTGGCTTGGACTTG-3’ |
|  |  | Reverse: 5’-GGTCACTCTGCTCGGTCGAACTA-3’ |
| β-actin | Human | Forward: 5’-TGGCACCCAGCACAATGAA-3’ |
|  |  | Reverse: 5’-CTAAGTCATAGTCCGCCTAGAAGCA-3’ |

Abbreviations: RT-qPCR, Real Time - quantitative Polymerase Chain Reaction; CYB5R3, Cytochrome B5 Reductase 3; CLIP4, CAP-Gly Domain Containing Linker Protein Family Member 4.

**Table S2.** 69 SARS-CoV-2 related differentially expressed genes.

| **Gene Symbol** | **Gene Symbol** | **Gene Symbol** | **Gene Symbol** |
| --- | --- | --- | --- |
| IL17RA | NUP58 | TM2D3 | NAT14 |
| SLC44A2 | FAM8A1 | FASTKD5 | FBLN5 |
| STOM | COMT | CLIP4 | GDF15 |
| TLE3 | NDUFB9 | DCTPP1 | JAKMIP1 |
| ITGB1 | HSBP1 | ERO1B | FKBP7 |
| CDK5RAP2 | GCC2 | TMEM39B | NDFIP2 |
| NUP214 | MEPCE | PLEKHA5 | HOOK1 |
| HMOX1 | NUTF2 | FOXRED2 | FKBP10 |
| PLD3 | SCARB1 | FBN2 | PLAT |
| CYB5R3 | ADAM9 | F2RL1 | PLOD2 |
| IMPDH2 | ERC1 | EXOSC5 | NINL |
| SCAP | SCCPDH | TMEM97 | CEP112 |
| WASHC4 | MFGE8 | FBN1 | STC2 |
| G3BP2 | NLRX1 | PCSK6 | TMPRSS2 |
| ATP13A3 | CTSL | CHPF | MARK1 |
| GPAA1 | POLA2 | ADAMTS1 |  |
| CHPF2 | PLEKHF2 | WFS1 |  |
| MAT2B | SIL1 | ZNF503 |  |

Abbreviations: SARS-CoV-2, Severe acute respiratory syndrome coronavirus-2.

**Table S3.** The results of functional enrichment analysis for SARS-COV-2 related DEGs.

| **GO terms** | | |
| --- | --- | --- |
| ID | Description | p adjust value |
| GO:0005788 | endoplasmic reticulum lumen | 0.0025 |
| GO:0062023 | collagen-containing extracellular matrix | 0.0126 |
| GO:0097493 | structural molecule activity conferring elasticity | 0.0014 |
| GO:0017056 | structural constituent of nuclear pore | 0.0129 |
| GO:0005178 | integrin binding | 0.0149 |
| GO:0005528 | FK506 binding | 0.0263 |
| GO:0004175 | endopeptidase activity | 0.0263 |
| GO:0005527 | macrolide binding | 0.0263 |
| GO:0001786 | phosphatidylserine binding | 0.0372 |
| GO:0005518 | collagen binding | 0.0464 |
| **KEGG and Reactome terms** | | |
| ID | Description | p adjust value |
| R-HSA-1474244 | Extracellular matrix organization | <0.0001 |
| R-HSA-2129379 | Molecules associated with elastic fibres | 0.0002 |
| R-HSA-1566948 | Elastic fibre formation | 0.0002 |
| R-HSA-1474228 | Degradation of the extracellular matrix | 0.0005 |
| R-HSA-1430728 | Metabolism | 0.0009 |
| R-HSA-8957275 | Post-translational protein phosphorylation | 0.0027 |
| R-HSA-381426 | Regulation of Insulin-like Growth Factor (IGF) transport and uptake by Insulin-like Growth Factor Binding Proteins (IGFBPs) | 0.0041 |
| hsa01100 | Metabolic pathways | 0.0090 |
| R-HSA-156581 | Methylation | 0.0140 |
| R-HSA-2022870 | Chondroitin sulfate biosynthesis | 0.0219 |
| **Metascape** | | |
| ID | Description | p adjust value |
| R-HSA-1474244 | Extracellular matrix organization | <0.0001 |
| R-HSA-9679506 | SARS-CoV Infections | <0.0001 |
| WP5038 | Mitochondrial immune response to SARS-CoV-2 | <0.0001 |
| WP4846 | SARS-CoV-2 and COVID-19 pathway | <0.0001 |
| R-HSA-2129379 | Molecules associated with elastic fibres | <0.0001 |
| GO:0033627 | cell adhesion mediated by integrin | 0.0001 |
| GO:0042060 | wound healing | 0.0001 |
| R-HSA-8957275 | Post-translational protein phosphorylation | 0.0001 |
| GO:0000226 | microtubule cytoskeleton organization | 0.0003 |
| GO:0001666 | response to hypoxia | 0.0005 |
| GO:0007034 | vacuolar transport | 0.0005 |
| GO:0030433 | ubiquitin-dependent ERAD pathway | 0.0008 |
| GO:0051223 | regulation of protein transport | 0.0012 |
| GO:0006457 | protein folding | 0.0015 |
| R-HSA-9679191 | Potential therapeutics for SARS | 0.0015 |
| GO:1901361 | organic cyclic compound catabolic process | 0.0016 |
| GO:1901137 | carbohydrate derivative biosynthetic process | 0.0023 |
| GO:0008203 | cholesterol metabolic process | 0.0025 |

Abbreviations: GO, Gene ontology; KEGG, Kyoto encyclopedia of genes and genomes; HSA, Homo sapiens; IGF, Insulin-like growth factor; IGFBPs, Insulin-like growth factor binding proteins; DEGs, Differentially expressed genes; SARS-CoV-2, Severe acute respiratory syndrome coronavirus-2; COVID-19, Corona virus disease-2019; ERAD, ER-associated degradation.

**Table S4.** Clinical characteristics of AML patients from UCSC Xena.

| **Aspects** | **TCGA-LAML (N=151)** |
| --- | --- |
| **Age at diagnosis** |  |
| >=60 years old | 68 (45.03%) |
| <60 years old | 83 (54.97%) |
| **Gender** |  |
| Male | 81 (53.64%) |
| Female | 70 (43.36%) |
| **Status** |  |
| Dead | 95 (62.91%) |
| Alive | 56 (37.09%) |
| **AML subtype** |  |
| M0 | 13 (8.61%) |
| M1 | 35 (23.18%) |
| M2 | 35 (23.18%) |
| M3 | 13 (8.61%) |
| M4 | 33 (21.85%) |
| M5 | 17 (11.26%) |
| M6 | 2 (1.32%) |
| M7 | 3 (1.99%) |
| **Neoadjuvant treatment** |  |
| Yes | 37 (24.50%) |
| No | 114 (75.50%) |
| **Cytogenetic risk** |  |
| Favorable | 29 (19.21%) |
| Intermediate/Normal | 89 (58.94%) |
| Poor | 31 (20.53%) |
| Missing | 2 (1.32%) |
| **Cytogenetic abnormalities** |  |
| Normal | 78 (51.65%) |
| Abnormal | 61 (40.40%) |
| Missing | 12 (7.95%) |
| **FLT3 mutation** |  |
| FLT3 Mut | 42 (27.81%) |
| FLT3 WT | 102 (67.55%) |
| Missing | 7 (4.64%) |
| **NPM1 mutation** |  |
| NPMc+ | 37 (24.50%) |
| NPMc- | 111 (73.51%) |
| Missing | 3 (1.99%) |

Abbreviations: UCSC, University of California Santa Cruz; TCGA, The Cancer Genome Atlas; AML, acute myeloid leukemia; FLT3, fms related receptor tyrosine kinase 3; NPM1, nucleophosmin 1.

**Table S5.** The results of Metascape for SC2RI in AML.

| **Top 20 pathways in Metascape** | | | |
| --- | --- | --- | --- |
| ID | Category | Description | Log10(P) |
| M5885 | Canonical Pathways | NABA MATRISOME ASSOCIATED | -18.21 |
| GO:0044057 | GO Biological Processes | regulation of system process | -12.77 |
| R-HSA-1474244 | Reactome Gene Sets | Extracellular matrix organization | -11.13 |
| M5884 | Canonical Pathways | NABA CORE MATRISOME | -10.81 |
| GO:0007610 | GO Biological Processes | behavior | -8.74 |
| GO:0050878 | GO Biological Processes | regulation of body fluid levels | -8.3 |
| GO:0032989 | GO Biological Processes | cellular component morphogenesis | -7.72 |
| GO:0071625 | GO Biological Processes | vocalization behavior | -7.65 |
| GO:0048732 | GO Biological Processes | gland development | -7.35 |
| GO:0009410 | GO Biological Processes | response to xenobiotic stimulus | -7.18 |
| GO:0003013 | GO Biological Processes | circulatory system process | -7 |
| GO:0008283 | GO Biological Processes | cell population proliferation | -6.89 |
| GO:0051051 | GO Biological Processes | negative regulation of transport | -6.8 |
| GO:0010469 | GO Biological Processes | regulation of signaling receptor activity | -6.79 |
| GO:0001501 | GO Biological Processes | skeletal system development | -6.74 |
| hsa04080 | KEGG Pathway | Neuroactive ligand-receptor interaction | -6.61 |
| GO:0031644 | GO Biological Processes | regulation of nervous system process | -6.57 |
| GO:0007389 | GO Biological Processes | pattern specification process | -6.17 |
| GO:0043408 | GO Biological Processes | regulation of MAPK cascade | -6.16 |
| GO:0042063 | GO Biological Processes | gliogenesis | -6.12 |
| **MCODE** |  |  |  |
| MCODE | ID | Description | Log10(P) |
| MCODE_1 | R-HSA-182971 | EGFR downregulation | -10.9 |
| MCODE_1 | R-HSA-177929 | Signaling by EGFR | -9.8 |
| MCODE_1 | hsa04022 | cGMP-PKG signaling pathway | -9 |
| MCODE_2 | R-HSA-500792 | GPCR ligand binding | -34.5 |
| MCODE_2 | R-HSA-373076 | Class A/1 (Rhodopsin-like receptors) | -34.1 |
| MCODE_2 | R-HSA-388396 | GPCR downstream signalling | -32 |
| MCODE_3 | R-HSA-1474244 | Extracellular matrix organization | -25.3 |
| MCODE_3 | R-HSA-216083 | Integrin cell surface interactions | -18.8 |
| MCODE_3 | hsa04512 | ECM-receptor interaction | -18.6 |
| MCODE_4 | WP702 | Metapathway biotransformation Phase I and II | -15.6 |
| MCODE_4 | hsa00591 | Linoleic acid metabolism | -9.6 |
| MCODE_4 | GO:0008610 | lipid biosynthetic process | -9.5 |
| MCODE_5 | R-HSA-6794361 | Neurexins and neuroligins | -6.7 |
| MCODE_5 | R-HSA-6794362 | Protein-protein interactions at synapses | -6.1 |
| MCODE_5 | R-HSA-114608 | Platelet degranulation | -5.6 |
| MCODE_6 | R-HSA-2129379 | Molecules associated with elastic fibres | -7.2 |
| MCODE_6 | R-HSA-1566948 | Elastic fibre formation | -7 |
| MCODE_6 | M3008 | NABA ECM GLYCOPROTEINS | -5 |
| MCODE_7 | R-HSA-6809371 | Formation of the cornified envelope | -11.1 |
| MCODE_7 | R-HSA-6805567 | Keratinization | -10 |
| MCODE_7 | GO:0045109 | intermediate filament organization | -9.3 |
| MCODE_8 | GO:0007156 | homophilic cell adhesion via plasma membrane adhesion molecules | -6.2 |
| MCODE_8 | GO:0098742 | cell-cell adhesion via plasma-membrane adhesion molecules | -5.6 |
| MCODE_8 | GO:0098609 | cell-cell adhesion | -4.7 |
| MCODE_9 | CORUM:6418 | C1q complex | -12.7 |
| MCODE_9 | R-HSA-173623 | Classical antibody-mediated complement activation | -11.4 |
| MCODE_9 | GO:0098883 | synapse pruning | -10.6 |

Abbreviations: SC2RI, SARS-COV-2 Risk Index; AML, Acute myeloid leukemia; MAPK, Mitogen-activated protein kinases; EGFR, Epidermal growth factor receptor; GPCR, G protein-coupled receptor; ECM, Extracellular matrix.

**Table S6.** The results of GSEA for SC2RI in AML.

| ID | Description | Enrichment Score | NES | p adjust value |
| --- | --- | --- | --- | --- |
| hsa04612 | Antigen processing and presentation | 0.540993934 | 1.726884386 | 0.029304953 |
| hsa05332 | Graft-versus-host disease | 0.642317386 | 1.79969134 | 0.016020478 |
| hsa05235 | PD-L1 expression and PD-1 checkpoint pathway in cancer | 0.511908348 | 1.725602729 | 0.016020478 |
| hsa04659 | Th17 cell differentiation | 0.529519448 | 1.807072923 | 0.012454878 |
| hsa04662 | B cell receptor signaling pathway | 0.531203209 | 1.747109376 | 0.016020478 |
| hsa04640 | Hematopoietic cell lineage | 0.503155694 | 1.699084878 | 0.027507647 |
| hsa04064 | NF-kappa B signaling pathway | 0.506439481 | 1.739676886 | 0.016020478 |
| hsa04660 | T cell receptor signaling pathway | 0.493311522 | 1.683507371 | 0.027507647 |
| hsa04672 | Intestinal immune network for IgA production | 0.598689647 | 1.748489939 | 0.027507647 |
| hsa04613 | Neutrophil extracellular trap formation | 0.422099512 | 1.548705951 | 0.029841273 |
| hsa04621 | NOD-like receptor signaling pathway | 0.419186242 | 1.52875991 | 0.032150721 |
| hsa04060 | Cytokine-cytokine receptor interaction | 0.394086657 | 1.502680624 | 0.029304953 |

Abbreviations: GSEA, Gene set enrichment analysis; SC2RI, SARS-COV-2 Risk Index; AML, Acute myeloid leukemia; NES, Normalized enrichment score; PD-1, Programmed cell death protein 1; PD-L1, Programmed cell death 1 ligand 1; Th17, T helper cell 17; NOD, Nucleotide-binding oligomerization domain.

**Table S7.** The results of drug sensitivity and SC2RI in CellMiner.

| SC2RI | Drug | Function | cor | pvalue |
| --- | --- | --- | --- | --- |
| SC2RI | Palbociclib | CDK4/6 inhibitors | -0.488667544 | <0.0001 |
| SC2RI | Oxaliplatin | antitumor drugs | -0.464981906 | 0.000181775 |
| SC2RI | By-Product of CUDC-305 | HSP90 inhibitors | -0.436779523 | 0.000484623 |
| SC2RI | OSI-027 | mTOR inhibitors | 0.412929749 | 0.001042105 |
| SC2RI | Volasertib | PLK1 inhibitors | -0.411797312 | 0.001079203 |
| SC2RI | Dexrazoxane | Adjuvant antitumor drugs | -0.403814972 | 0.001376273 |
| SC2RI | Tamoxifen | Estrogen receptor antagonists | -0.39830202 | 0.001622337 |
| SC2RI | GSK-2126458 | mTOR inhibitors | 0.39480746 | 0.001798045 |
| SC2RI | Des-fluoro-TAK-960 | PLK1 inhibitors | -0.375375577 | 0.003122844 |
| SC2RI | JNJ-38877605 | c-Met inhibitors | 0.370385698 | 0.003579534 |
| SC2RI | Ifosfamide | antitumor drugs | -0.365354748 | 0.004098914 |
| SC2RI | AFP464 | HIF-1α inhibitors | -0.354577896 | 0.005440729 |
| SC2RI | Belinostat | antitumor drugs | -0.352833148 | 0.005690918 |
| SC2RI | Ribavirin | antiviral agent | -0.346447294 | 0.006694876 |
| SC2RI | Barasertib | Aurora B inhibitors | -0.336251565 | 0.008619943 |
| SC2RI | Deforolimus | mTOR inhibitors | 0.33401538 | 0.009101424 |
| SC2RI | Simvastatin | hypolipidemic drugs | 0.333072781 | 0.00931128 |
| SC2RI | CUDC-305 | HSP90 inhibitors | -0.332660101 | 0.009404473 |

Abbreviations: SC2RI, SARS-COV-2 Risk Index.
